# Late Miocene Leaves and Endocarps of *Choerospondias* (Anacardiaceae) from Zhejiang, Eastern China: Implications for Paleogeography and Paleoclimate

**DOI:** 10.3390/biology11101399

**Published:** 2022-09-25

**Authors:** Liang Xiao, Zeling Wu, Liyan Guo, Xiangchuan Li, Deshuang Ji, Xiaoyuan Xia, Jianan Wang, Jiaqi Liang, Nan Sun

**Affiliations:** 1 School of Earth Science and Resources, and Key Laboratory of Western Mineral Resources and Geological, Engineering of the Ministry of Education, Chang’an University, Xi’an 710054, China; 2 State Key Laboratory of Palaeobiology and Stratigraphy, Nanjing Institute of Geology and Paleontology, Chinese Academy of Sciences, Nanjing 210008, China; 3 Shaanxi Key Laboratory of Early Life and Environments, Northwest University, Xi’an 710069, China

**Keywords:** *Choerospondias*, late Miocene, paleogeography, paleoclimate, eastern Zhejiang, China

## Abstract

**Simple Summary:**

*Choerospondias* endocarp and leaf fossils were found in the Shengxian Formation of Zhejiang, eastern China. We performed cuticle analysis on leaves and micro-CT on endocarps to reconstruct three-dimensional morphological characteristics. Fossil records suggest that *Choerospondias* spread from low to middle latitudes throughout geological time, and migrated to the northern boundary of the distribution range in China during the late Miocene. Based on the climatic parameters under which extant *Choerospondias* live, the paleoclimatic values of eastern Zhejiang in the late Miocene were obtained and compared with previously published paleoclimatic data. The results suggest that the climate of the late Miocene in the Tiantai region of Zhejiang was warm and humid, similar overall to the modern climate of this region.

**Abstract:**

*Choerospondias* (Anacardiaceae), characterized by radially arranged germination pores near the top, is a monotypic genus mainly distributed in subtropical and tropical eastern Asia, while fossil records indicate a wide distribution throughout Eurasia during the Cenozoic. In this study, we reported three-dimensionally preserved *Choerospondias* endocarps, and the associated compressed leaves from the late Miocene Shengxian Formation in Tiantai, Zhejiang, eastern China. The plant remains were assigned to two new fossil species. The endocarps were identified as *Choerospondias* *tiantaiensis* sp. nov., and the leaves were identified as *Choerospondias mioaxillaris* sp. nov. Based on fossil records and climate fluctuation during the Cenozoic, we conclude that *Choerospondias* may have originated from Europe in the early Eocene and then spread to Asia along the coast and island chains of the Tethys and Paratethys oceans. The distribution position of the current fossils was adjacent to the northern boundary of the modern distribution of *Choerospondias* in East Asia, indicating that the distribution pattern of *Choerospondias* in East Asia likely formed no later than the late Miocene. We reconstructed the late Miocene paleoclimate of eastern Zhejiang by using the method of climate analysis of endemic species (CAES), and then compared it to the data reconstructed in previous studies. The results indicate that the late Miocene climate in eastern Zhejiang was similar to or warmer and more humid than the modern climate in this region.

## 1. Introduction

**Figure 1:**
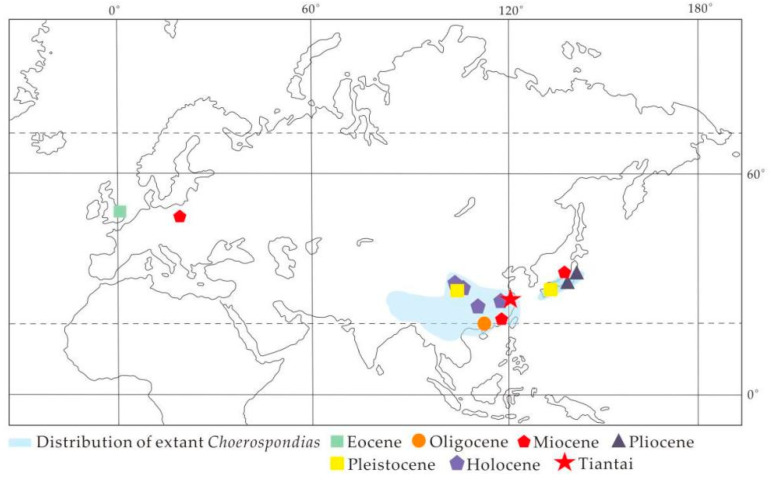
Distribution of extant and fossil *Choerospondias* (modified from [18]). The extant distribution of *Choerospondias axillaris* in Asia is shown in the light blue area. The symbols indicate fossil sites of *Choerospondias* in different geological ages. The red asterisk represents the fossil site in eastern Zhejiang.

*Choerospondias*, which belongs to the subfamily Spondioideae in the Anacardiaceae, is a deciduous tree [1,2]. This genus is monotypic—*C. axillaris* (Roxb.) Burtt et Hill. It is mainly distributed in southern Japan and south of the Yangtze River in China, as well as in the northern part of the Indochina Peninsula and the Eastern Himalayan region (Figure 1), while the hirsute variety *C. axillaris* var. *pubinervis* is distributed in eastern Sichuan, southern Gansu, western Hubei, and western Hunan [1].

Only seven macrofossil records of *Choerospondias* have been previously reported. All are endocarp fossils distributed in Europe and East Asia. In addition, leaf fossils have not been reported. There are two fossil records in Europe: the first is *C. sheppeyensis*, which is the earliest fossil record from early Eocene London, southern England [3,4,5], while the other is the middle Miocene *C. turovensis* from Turow, Poland [6]. In East Asia, three samples are from Japan, including two fossils of *C. axillaris* (Roxb.) Burtt et Hill. from late Miocene and Pliocene Honshu [7,8,9] and one fossil of *Choerospondias.* sp. cf. *C. axillaris* from the Pliocene [10,11]. Two fossils are from China, including *C. nanningensis* from late Oligocene Nanning, Guangxi [12], and *C. fujianensis* from middle Miocene Fotan, Fujian [13], both of which are located at low latitudes near the Tropic of Cancer. Among these fossil endocarps, *C. nanningensis* has the lowest latitude. *Choerospondias* endocarps have also been found at several archaeological sites in China corresponding to the Quaternary period, indicating that early humans living in these areas used *Choerospondias* as a food source [13,14,15,16,17]. Previous studies on *Choerospondias* fossils tend to focus on taxonomy and paleoecology, while the origin and dispersal pathways of the *Choerospondias* remain ambiguous.

In this research, two fruit fossils and three leaf fossils were collected simultaneously from the Shengxian Formation, eastern Zhejiang, China. Micro-CT and microtomy technology were used to observe the whole three-dimensional morphology. Based on these, the fossilized endocarps of *Choerospondias* from the Shengxian Formation were reliably identified as a new species. In addition, the fossil leaves of *Choerospondias* were investigated for the first time. According to the leaf architecture and cuticular features, the leaf remains were also classified into a new species. Furthermore, the biogeographic dispersal of the genus was inferred based on the fossil records of *Choerospondias*. Finally, according to the climatic parameters under which extant *Choerospondias* live, the paleoclimate of eastern Zhejiang in the late Miocene was reconstructed and further compared to previously obtained values.

## 2. Materials and Methods

### 2.1. Geological Setting

**Figure 2:**
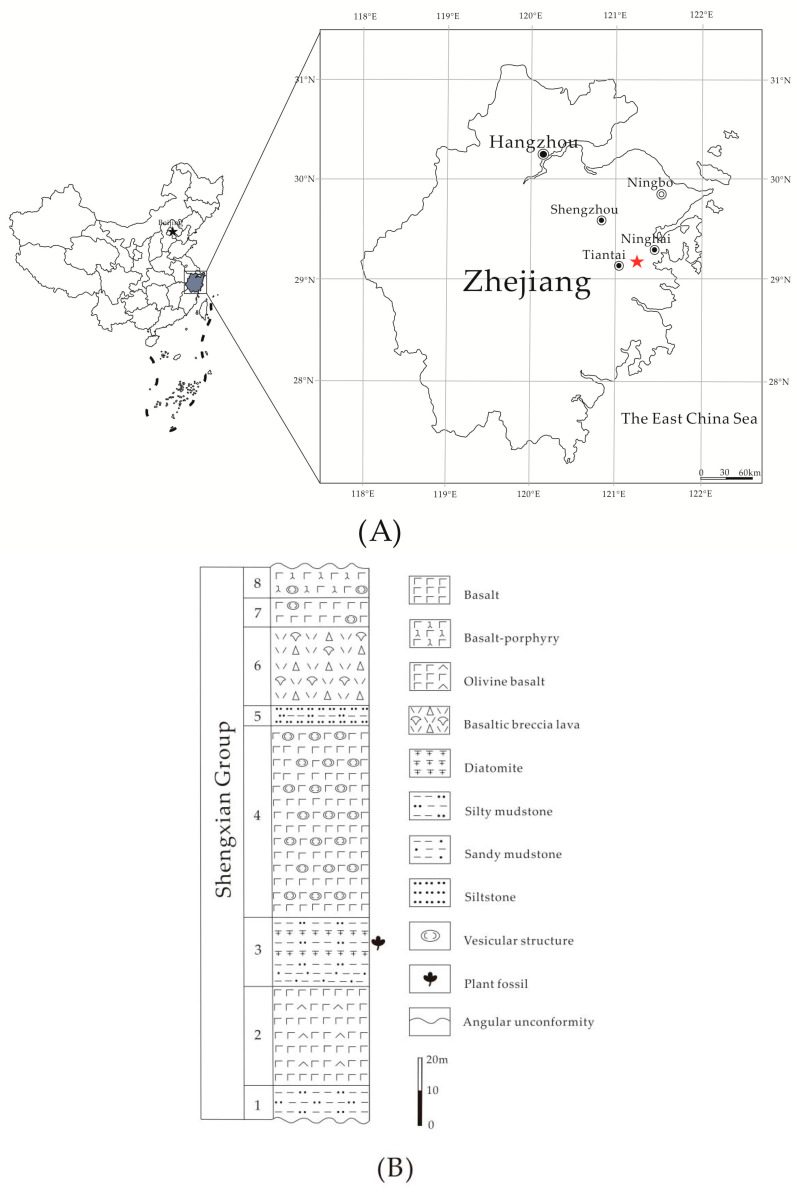
(**A**) Fossil site (red pentagram) of present fossil. (**B**) Stratigraphic column of the Shengxian Formation in eastern Zhejiang, China.

The *Choerospondias* fossils studied here were collected from the Shengxian Formation (29°09′ N, 121°14′ E) in eastern Zhejiang, China. The lithology of the Shengxian Formation is composed of a set of continental volcanic basalt intercalated sedimentary layers that consist of dark-gray or gray fluvial-lacustrine diatomite mudstone, siltstone, sandstone, and a thin lignite layer, forming a unique multicyclic stratigraphic sequence of basalt and clastic rock [19] (Figure 2). Ho et al. [20] and Wang et al. [21] dated the formation to 13.0~10.5/9.4 Ma (^40^Ar-^39^Ar method) or 13.00~10.38 Ma (K-Ar method). He [22] determined the age to be 13.0~6.0 Ma, i.e., the middle Miocene to late Miocene, using the ^40^Ar/^39^Ar dating method. Using stratigraphic correlation [23] and comparison [24,25,26], the geological age of the Shengxian Formation was confined to the late Miocene. There are abundant plant fossils preserved in the intercalated sedimentary layers of the Shengxian Formation. The fossil plants are mainly composed of angiosperms and a few conifers and rare ferns [19,24,25,26,27,28,29,30,31].

### 2.2. Material Preparation

The two fruits and three leaves studied in this paper were collected from the late Miocene Shengxian Formation in eastern Zhejiang, China. The specimens of living *Choerospondias* were collected from the Hangzhou Botanical Garden in July 2021. All the extant and fossil *Choerospondias* specimens are stored in the Geological Museum of Chang’an University.

#### 2.2.1. Fossil Preparation

For the treatment of endocarp fossils, the surfaces of the fossils were first wiped with anhydrous ethanol and then photographed under a VHX-1000 super depth-of-field microscope. Next, we used the ZEISS Xradia 520 Versa 3D X-ray microscope at Northwest University to obtain raw CT data on the internal structure of the endocarp fossils. The data were then processed using the Visual Studio MAX 3.0 software (Volume Graphics, Germany) to obtain virtual sections. According to these processed data, the 3D structures of the fruit fossils were accurately reconstructed. In addition, transversal sections were obtained for a conventional anatomic study using the epoxy resin embedding method [32]. They were subsequently observed and photographed under a Leica DM1000 optical microscope.

For the treatment of leaf fossils, the fossils were photographed with a Leica M165FC stereomicroscope. The cuticular analysis was performed according to the procedure described by Liang et al. [33]. The fossils were moistened well in distilled water and then soaked in 20% HCl solution for 12 h to remove calcareous sediments; then, they were washed to neutrality using distilled water. Subsequently, they were soaked in HF solution (40%) for 12 h to remove siliceous sediments and then washed again with distilled water until neutral. Next, they were transferred into 70% HNO_3_ reagent and immersed for 4–12 h. When the color of the cuticles changed from black to transparent light brown, the samples were taken from the solution and washed with distilled water. Next, the cuticles were immersed in distilled water for 1–2 h to fully remove any residual HNO_3_ in the cuticle. Following this procedure, the leaves were soaked in 0.4% NaClO solution for 5–10 s and observed under a dissecting microscope until the leaf edges showed signs of epidermal separation, at which point they were washed to neutrality with distilled water. The cuticles were stained with 1% safranin solution for less than 10 s, depending on the thickness of the leaf fossil, and then mounted. Finally, they were photographed under a Leica DM1000 optical biomicroscope.

The terminology used for describing the endocarp fossils follows that of Fu et al. [12] and Herrera et al. [34]. The terminology used for describing the leaf fossils follows that of Li et al. [35] and Wang et al. [36].

#### 2.2.2. Extant Leaf Cuticle Treatment

A small piece of leaf from extant *Choerospondias* was cut off and placed into a beaker with a mixed solution containing 30% H_2_O_2_ and 99% CH_3_COOH (1:1 *v*/*v*) before being transferred into a water bath kettle kept at 70 °C for approximately 6 h. When the leaf became transparent with the occurrence of bubbles, it was washed to neutrality using distilled water. The upper and lower epidermis were peeled off using dissecting needles and tweezers. Subsequently, the epidermis was gently brushed to remove the adhered mesophyll. The clean upper and lower epidermis were stained with 1% safranin solution for approximately 1 min and then mounted. Finally, they were photographed under a Leica DM1000 optical biomicroscope. 

## 3. Systematic Paleobotany

Order: Sapindales Juss. ex Bercht. et J. Presl.

Family: Anacardiaceae R. Br. 

Subfamily: Spondiadoideae Kunth ex Arn.

Genus: *Choerospondias* Burtt et Hill.

Species: *Choerospondias tiantaiensis* Liang Xiao et Zeling Wu sp. nov.

Etymology: The epithet refers to Tiantai County, where the specimens were collected.

**Figure 3:**
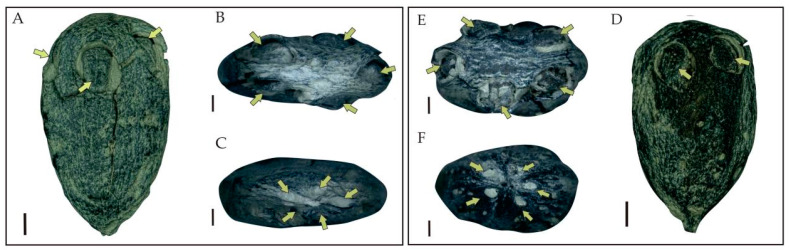
Characteristics of endocarps of *Choerospondias tiantaiensis* under a VHX-1000 super-depth-of-field microscope ((**A**–**C**), JHU3-16-059; (**D**–**F**), JHU3-231). (**A**,**D**): Lateral view of endocarp showing the germination pores (yellow arrows). (**B**,**E**): Top view of endocarp showing the five germination pores (yellow arrows). (**C**,**F**): Base view of endocarp showing the five apertures in a whorl (yellow arrows). (**A**,**D**), scale bar = 2 mm. (**B**,**C**,**E**,**F**), scale bar = 1 mm.

**Figure 4:**
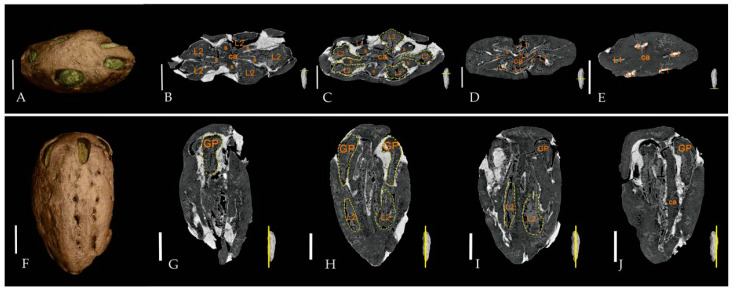
Characteristics of endocarps of *Choerospondias tiantaiensis* under micro-CT. ((**A**–**J**), JHU3-16-059) (**A**,**F**): micro-CT volume renderings. (**B**–**E**,**G**–**J**): micro-CT orthoslices of endocarp showing the internal structures. s = septa; ca = central axis; L1 = lacunae; L2 = locules; GP = germination pore. (**B**–**E**): Transverse sections of fossil endocarp from the top to the base, showing the arrangement of locules (yellow-dotted lines) and lacunae through the long axis of the endocarp. (**G**–**J**): Longitudinal sections of fossil endocarp showing the arrangement of locules (yellow-dotted lines) and germination pores. (**A**–**E**), scale bar = 2 mm; (**G**–**J**), scale bar = 3.5 mm.

Holotype: JHU3-16-059 (Figure 3A–C and Figure 4A–J).

Paratype: JHU3-231 (Figure 3D–F).

Locality: Jiahu Village, Tiantai County, Zhejiang Province, China (29°09′ N, 121°14′ E, Figure 2).

Stratigraphy: The Shengxian Formation.

Age: Late Miocene.

Repository: Geological Museum of Chang’an University, Chang’an University, Xi’an, China.

Diagnosis: The endocarps were ovoid to oval with obscure ridges on their surface and some irregularly arranged pits. Five germination pores were observed distributed radially near the top. Five pits radially situated around the subacute base of the outer endocarp were also observed. The endocarp wall was composed of irregularly arranged fibers and smaller oval-shaped isodiametric sclereids. A clear boundary existed between the nexine and endothecium of the endocarp. 

Description: 

External features of endocarps—The endocarps were black, woody, slightly compressed, ovoid to oval in shape, 16.4–20 mm long, and 9.8–11.7 mm wide, with length–width ratios ca. 1.7. Five inconspicuous ridges were arranged longitudinally along the longitudinal axis of the outer surface of the endocarp. A large number of pits were loosely arranged on both sides of each ridge. There were five germination pores in each endocarp, which were arranged radially near the top. Each pore corresponded to a locule. A corresponding number of inconspicuous longitudinal ridge arrangements were observed on the surface of the endocarp. The shapes of the germination pores were obovate to elliptical with a diameter of 3.6–4.7 mm (Figure 3).

Internal features of endocarps—The endocarps were oblong when viewed in the transversal sections and were possibly compressed during diagenesis. There were five locules radially arranged inside. All locules in each fruit fossil collected in this research were well-developed. In the transversal sections, the five locules had ovoid to elliptical shapes and were 1.6–2.1 mm long and 1.1–2.0 mm wide (Figure 4B–E). In the longitudinal sections, the endocarps had a long oval shape, 4.5–7.8 mm long and 1.5–2.7 mm wide (Figure 4G–J). Five septa were arranged radially on the medial axis with round to long teardrop shapes (probably due to compression). The locules were distributed on both sides of the septa. Five irregular pores were observed in a radial arrangement in the cross section near the bottom (Figure 4B–E).

**Figure 5:**
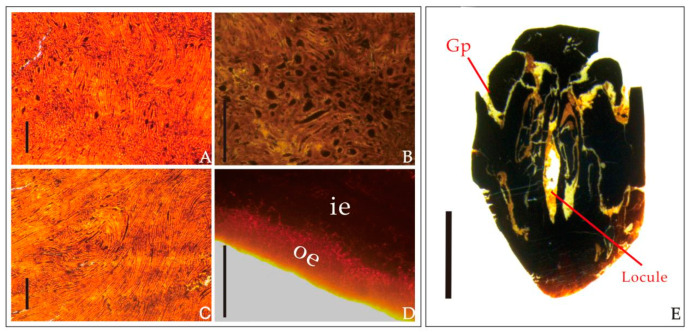
Internal structure and anatomical details of the *Choerospondias tiantaiensis* sp. nov. fossil endocarp ((**A**–**E**), JHU3-16-059). (**A**): Fibers and sclereids of the endocarp wall, scale bar = 0.1 mm. (**B**): Details of sclereids, scale bar = 0.1 mm. (**C**): Fibers structure in the septum, scale bar = 0.1 mm. (**D**): Irregular arrangement of fibers outside the endocarp and closely arranged sclereids inside the endocarp in transmitted light. Scale bar = 0.1 mm; oe, outer endocarp; ie, inner endocarp. (**E**): Longitudinal section showing the overall structure of the endocarp, locules, and germination pore (Gp). Scale bar = 5 mm.

Anatomical features of endocarps—The endocarp wall was composed of irregularly arranged fibers and smaller oval-shaped isodiametric sclereids (Figure 5A–C). Abundant and obvious dark filler was observed in the space between the germination pore and the locules. The dark inner nexine of the endocarp was arranged tightly and irregularly. However, the arrangement of fibers in the endothecium of the endocarp was loose and lighter in color. Thus, a clear boundary between the nexine and endothecium of the endocarp was easily observed (Figure 5D).

Comparison:

*Choerospondias* fruit is a drupe type, which is oval or obovate–oval. Its outer layer is woody. The germination pores are arranged radially on the top of the inner layer. Each locule in the germination pore contains a single seed [1,34]. The endocarp fossils in this study possessed the distinct characteristics of *Choerospondias* mentioned above. Thus, it was uncontroversial to classify the present fossil endocarps as *Choerospondias*.

*Choerospondias* fossils have been reported from the early Eocene to the Pleistocene strata [3,4,5,6,8,12,13,37,38]. The differences between those fossil species are reflected in their size, number of germination pores, and internal anatomical characteristics. The sizes of the present fossil endocarps were 16.4–20 mm × 9.8–11.7 mm, while those of the early Eocene *C. sheppeyensis* fossils from England and middle Miocene *C. turovensis* fossils from Poland were 12–13 mm × 11–12.5 mm [3,4,37] and 9.5–12 mm × 5–9 mm [6], respectively. It was shown that the two *Choerospondias* fossils from Europe were significantly smaller than the Zhejiang fossils. Among the Asian *Choerospondias* fossil endocarps, the Pliocene *C.* sp. cf. *C. axillaris* [38] endocarp fossil from Japan was large (45 mm × 23 mm), almost twice as large as the Zhejiang fossil. Because of the difference in size, the possibility of identifying the fossil as *C.* sp. cf. *C. axillaris* was also ruled out. Additionally, all three *Choerospondias* fossils mentioned above lacked the anatomical characteristic of the endocarp, which prevented further micromorphological comparison.

The size of the *Choerospondias fujianensis* fossils from middle Miocene Fujian was 15.7–21.4 mm × 15.7–20.5 mm, larger than that of the Zhejiang fossils (16.4–20 mm × 9.8–11.7 mm) [13]. Moreover, *C. fujianensis* appeared to have seven pores, while the Zhejiang fossils only had five pores. Furthermore, the boundary between the inner and outer layers of the *C. fujianensis* endocarp was indistinct, different from clear boundary of the Zhejiang fossils. The size of the late Oligocene *C. nanningensis* fossils from Guangxi was slightly larger (15–21 mm × 13–17 mm) than that of the Zhejiang fossils [12]. However, the boundary between the inner and outer layers of the *C. nanningensis* endocarp was also blurred, again different from that of the Zhejiang fossils. Therefore, the Zhejiang fossils considered in this research could not be classified as any known fossil species.

The endocarp of Zhejiang fossils is similar to that of extant *Choerospondias axillaris* in sunken bilabial germination structure, the number of germination pores, and the corresponding pits at the base of the endocarps. Symmetrically distributed internal lacunae and locules of Zhejiang fossils, along with other anatomical features, were consistent with those of the extant *Choerospondias* endocarp (Table 1). For external morphology, the endocarp fossils from Zhejiang (16.4–20 mm × 9.8–11.7 mm) were smaller than the extant endocarp (21–25 mm × 14–17 mm). The difference in size between the fossilized and extant endocarp may have been due to compression and dehydration during fossilization. However, due to the absence of other organs, such as flowers, as well as the remote gap between the late Miocene and today (approximately 13–6 Ma), we treated the fossil endocarps as a new species, *C. tiantaiensis*. 

Order: Sapindales Juss. ex Bercht. et J. Presl.

Family: Anacardiaceae R. Br. 

Subfamily: Spondiadoideae Kunth ex Arn.

Genus: *Choerospondias* Burtt et Hill.

Species: *Choerospondias mioaxillaris* Liang Xiao et Zeling Wu sp. nov.

Etymology: The epithet refers to the Miocene *Choerospondias axillaris*, indicating that the present fossil leaf resembles the extant *Choerospondias* in morphology.

**Figure 6:**
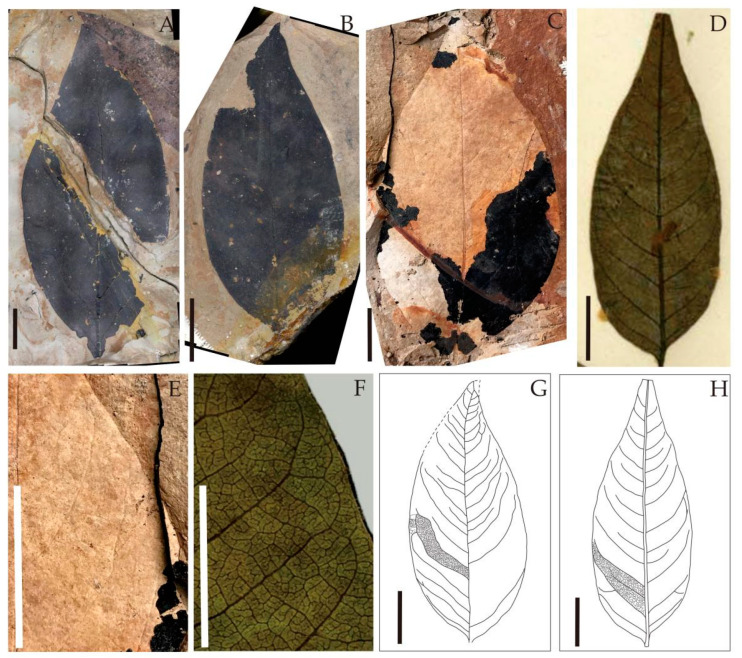
Leaf architecture of the extant and fossil *Choerospondias* ((**A**), GT-14-629; (**B**), GT-14-468; (**C**), DLC-14-100A). (**A**–**C**): Fossil *Choerospondias*. (**D**): Extant *Choerospondias*. (**E**): Leaf margin of fossil *Choerospondias*. (**F**): Leaf margin of the extant *Choerospondias*. (**G**): Sketch of fossil *Choerospondias.* (**H**): Sketch of the extant *Choerospondias*. Scale bar = 1 cm.

Holotype: DLC-14-100A (Figure 6C).

Paratype: GT-14-629 (Figure 6A), GT-14-468 (Figure 6B).

Locality: Jiahu Village, Tiantai County, Zhejiang Province, China (29°09′ N, 121°14′ E, Figure 2). 

Stratigraphy: The Shengxian Formation. 

Age: Late Miocene. 

Repository: Geological Museum of Chang’an University, Chang’an University, Xi’an, China.

Diagnosis: Leaves ovate to ovate-lanceolate, apex shape long and acuminate, base shape convex slightly. The leaf margin was entire. Primary venation pinnate, Secondaries brochidodromous. Secondary angles to midvein 40–60°. Intersecondary vein present. The basal veins of the intersecondary veins parallel to the secondary veins. Epidermal cells were irregular and polygonal in shape, anticlinal walls undulated shallowly. Trichome multicellular. Stomata cyclocytic.

Description: 

Leaf architecture—The leaves were ovate to ovate-lanceolate, 5–8.5 cm long, and 2–3.6 cm wide. The leaf margin was entire. The leaf base was slightly convex, with an angle of approximately 100–119°, and no petiole was preserved. The leaf apex was long and acuminate and the angle was about 53°. The venation was brochidodromous. The secondary veins—pinnately arranged, 10 pairs—were found to rise from the primary vein at an angle of approximately 40–60°, gradually becoming thinner near the leaf margin, with the basal veins of the intersecondary veins parallel to the secondary veins (Figure 6A–C,E,G).

**Figure 7:**
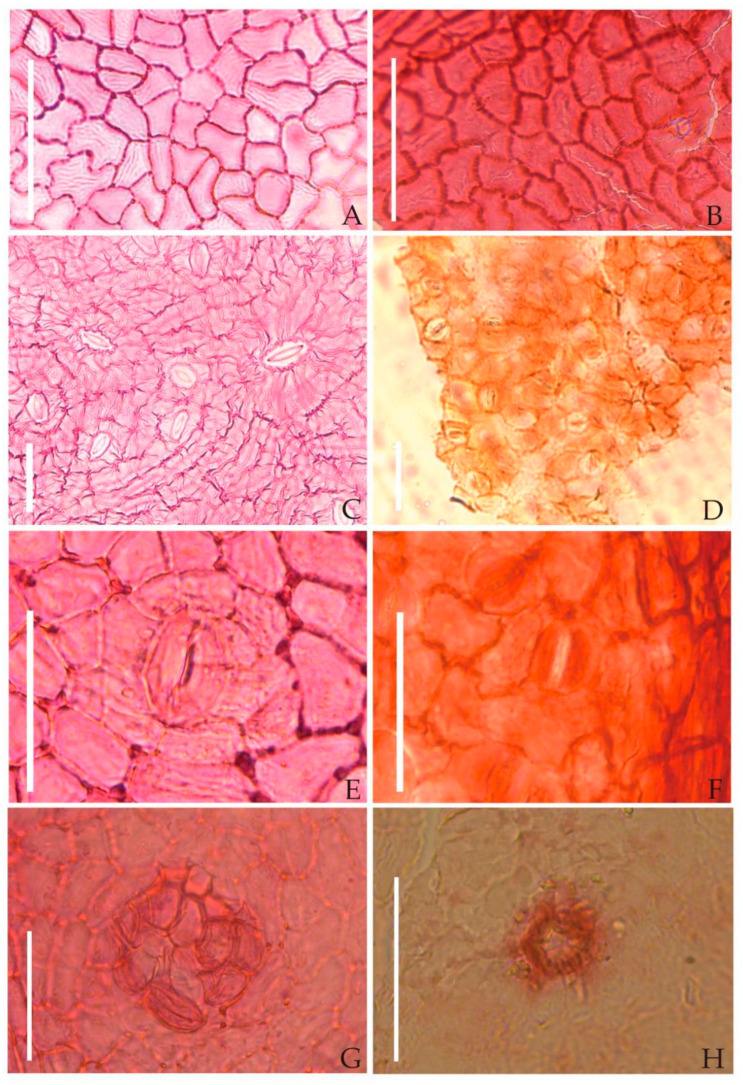
Epidermal characteristics of the extant and fossil *Choerospondias* ((**B**,**D**,**F**,**H**), GT-14-629). (**A**): Epidermal cells in the upper epidermis of the extant *Choerospondias*. (**B**): Epidermal cells in the upper epidermis of fossil *Choerospondias*. (**C**): Lower epidermal of extant *Choerospondias*. (**D**): Lower epidermal of fossil *Choerospondias*. (**E**): Lower epidermal stomatal apparatus of the extant *Choerospondias*. (**F**): Lower epidermal stomatal apparatus of fossil *Choerospondias.* (**G**): Extant *Choerospondias* trichome base. (**H**): Fossil *Choerospondias* trichome base. (**A**,**B**): Scale bar = 0.1 mm; (**C**–**H**): scale bar = 0.05 mm.

Leaf cuticle characteristics—The upper epidermal cells were irregular and polygonal, varying from quadrilateral to hexagonal. The anticlinal walls were shallowly undulated. The lower epidermal cells were also irregular and polygonal. The stomatal apparatus was 15–35 μm long and 10–30 μm wide, cyclocytic and randomly distributed. The trichome bases were multicellular (Figure 7).

Comparison: 

Here, fossilized *Choerospondias* leaves were compared to their living equivalents for the first time. The fossils investigated in the current study resemble the extant *Choerospondias* in size as well as in leaf shape, basal and apical morphology, and especially vein architecture. For the extant *Choerospondias* leaves, the angle between the secondary veins and the midrib was approximately 50–60°, which enlarged from the apex to the base. The tertiary veins were unbranched and almost perpendicular to the secondary veins. On the other hand, the secondary veins of the Zhejiang leaf fossils possessed a relatively uniform angle that intersected the midrib at 40–60°. After a detailed comparison, it was concluded that the difference in leaf architecture between the fossil and extant *Choerospondias* leaves was small.

In this research, we further obtained the cuticular microstructure of fossilized *Choerospondias* leaves. The stomatal apparatus was found to be cyclocytic. A few multicellular trichome bases were observed on the epidermis, distributed mainly on the leaf veins. These identified features are consistent with those of extant *Choerospondias* leaves, as described by Zheng et al. [2]. In addition, other epidermal characteristics, including cell shape, anticlinal walls, and venation cell shape, were also similar between the fossilized and living *Choerospondias* leaves (Table 2). However, due to the remote gap between the late Miocene and today, we treated the fossil leaves as a new species, *Choerospondias mioaxillaris* sp. nov.

Although the leaf specimens and co-occurring endocarps were collected from the same layer of the same site in Tiantai and seem to belong to the same species, they were not directly linked together. For the sake of caution, we did not assign the two fruit fossils and the three leaf fossils to the same species. Overall, the morphologies of both the endocarps and leaves of late Miocene *Choerospondias* in eastern Zhejiang were more similar to their living relatives. This implies that the present fossil species of *Choerospondias* may be the ancestor of extant *C. axillaris.*

## 4. Discussion

### 4.1. Paleogeographic History of Choerospondias

The reviewed fossil records of *Choerospondias* are restricted to East Asia and Europe [3,4,5,6,8,12,13,37,38]. To date, the most ancient *Choerospondias* plants were reported in Western Europe from the early Eocene [5]. Since the late Oligocene, *Choerospondias* has spread to East Asia [12]. During the Miocene, this genus existed in Eastern Europe and East Asia. However, *Choerospondias* has been confined to East Asia since the Pliocene (Figure 1; Table 1). Based on these fossil records, it could be supposed that *Choerospondias* plants may originate from Europe in the early Eocene, whereafter they spread to Asia before the late Oligocene. During the Paleogene and Neogene, there were two possible pathways for *Choerospondias* plants to spread from Europe to Asia. One was the northern route at middle-to-high latitudes, which stretched across the Turgai region, north of the Tibetan Plateau [39,40,41]. The other was the southern route, which crossed along the southern border of the Tibetan Plateau via the coast and the island chains of the Tethys and Paratethys oceans [42,43,44,45,46].

The Turgai region was flooded by the epicontinental Turgai sea during most of the Palaeogene and fell dry at the end of the Oligocene. Henceforth, the Turgai region became an important pathway for biological migration between Europe and Asia [39]. However, with the closure of the Turgai Strait, Central Asia became increasingly cool and dry following the late Paleogene [47,48,49,50,51]. During the early Oligocene, the flora in the Turgai region was of the temperate forest type [52,53]. By the late Oligocene, forest-steppe flora was established in this region [54,55,56,57,58]. Taken together, the above paleoclimatic and paleofloral reports and reconstructions indicate that a temperate climate was predominant in the Turgai region during the late Oligocene into the middle Neogene. However, *Choerospondias* fossils from many regions in Central Europe and Asia during this period were reported to show fully humid tropical to warm temperate character [13]. The climatic preferences of extant and fossil *Choerospondias* point toward a migration route with warm and humid conditions. Thus, the northern route was not suitable for the spread of *Choerospondias*.

Compared with the Turgai region, the Tethys ocean lay at lower latitude and had warmer and wetter climate, which allowed for tropical and subtropical plants to live along the coast [39,46,59], by providing natural conditions for the survival and migration of tropical and subtropical plant taxa on the Eurasian continent. Under the influence of the collision between the Eurasian and African/Indian plates, the Tethys and Paratethys oceans had been gradually closing since the Eocene [60]. Simultaneously, with the formation of Antarctic glaciers in the late Eocene, the sea level decreased by approximately 50–60 m, leading to the formation of a larger land area in the Tethys and Paratethys ocean regions [60,61]. Ultimately, these two factors expanded the territory available for the biological spread on the continent of Eurasia [62,63,64]. In addition, many large adjacent islands and peninsulas (e.g., Anatolia) existed in the Tethys and Paratethys oceans, which would also have provided possible pathways for biotic migration across narrow straits [18,42,49,65,66]. For example, Bowerbank [67] reported that *Nipa* fossils, i.e., a tropical palm, spread to Southeast Asia along the Tethys Ocean from Britain across Eurasia [3,68,69,70]. The earliest fossil of *C. nanningensis* from East Asia was reported in late Oligocene Nanning, Guangxi [12], indicating that the *Choerospondias* plant may have first migrated from Europe to Southern Asia along the southern route before the late Oligocene. However, after the middle Miocene, *Choerospondias* fossils disappeared in Europe due to continuous global cooling during the late Cenozoic. Consequently, the *Choerospondias* population may have been unable to adapt to climatic change, finally leading to extinction in Europe [6,71].

**Figure 8:**
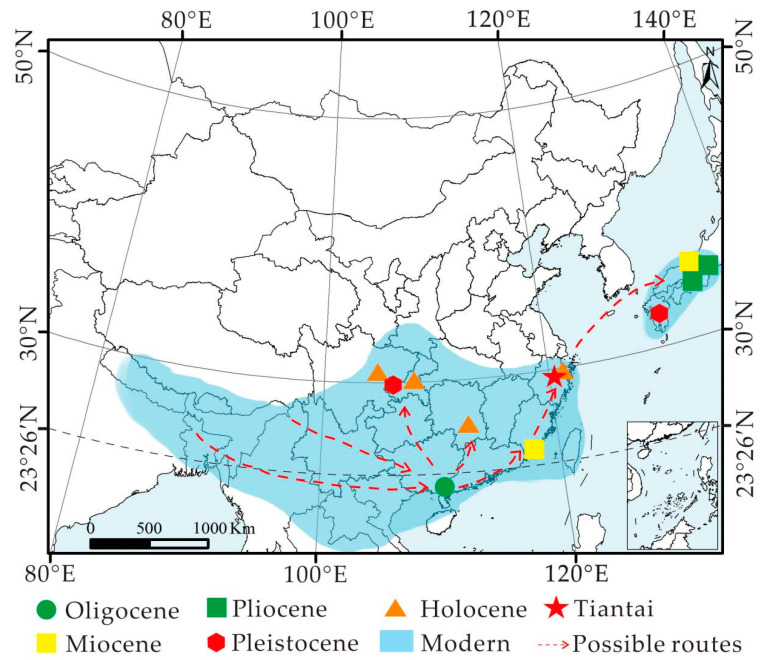
The fossil records and the present distribution range (representing in blue color) of *Choerospondias* in Asia. The red dotted line represents the dispersal route of *Choerospondias* in Asia.

The distribution of *Choerospondias* fossils in East Asia is concentrated in southeastern China and southern Japan. The late Oligocene *C. nanningensis* in Nanning, Guangxi is currently the earliest fossil record in East Asia with the lowest latitudinal distribution. According to all the fossil records of this genus in China, the latitudinal distribution of *Choerospondias* is getting higher over geological time (Figure 8). It was speculated that *Choerospondias* plants in China showed a trend of gradually northward spread from low to high latitudes, which could be related to the paleoclimatic fluctuation in East Asia. During the Miocene, global temperatures were higher relative to the present [72,73], and the monsoonal circulation system had formed [74,75,76,77], causing a warm and humid climate in eastern China [51,78]. This provided favorable climatic conditions for *Choerospondias* populations to live in southeastern China.

The distribution position of the fossils in the current study is consistent with the northern boundary of the distribution of living *Choerospondias* plants in eastern China, suggesting that the distribution pattern of this genus in China formed no late than the late Miocene, possibly due to cold and dry conditions after the late Miocene (ca. 8 Ma) [42,79]. The climatic change prevented the genus from spreading to higher latitudes. In contrast, the beneficial topography and climate in South and East China provided a relatively stable refuge, which contributed to the survival of the *Choerospondias* taxon in the cold climate of the Quaternary ice age [12,13,66,80].

The earliest fossil record of *Choerospondias* in Japan is *C. axillaris* from the late Miocene Osaka, southern Japan. The same *Choerospondias* plants have been found in the area until today [8,38]. The latitudinal distribution of *C. axillaris* was the highest in Japanese fossils until now, adjacent to the northern boundary of the living *Choerospondias* distribution in Japan (Figure 8), which indicates that the distribution pattern of *Choerospondias* in Japan was roughly formed since the late Miocene. This may have been due to southern Japan’s specific topography and oceanic climate, which helped *Choerospondias* live in a cold climate during the Quaternary ice age in Japan, and then survive until now. The distribution pattern of *Choerospondias* in Japan was similar to that in China, suggesting that phytogeographic change was consistent in eastern Asia after the Miocene. 

The fossil records of *Choerospondias*, however, are not sufficient. It is still difficult to accurately infer its geographic origin and detailed dispersal process over geological time. Thus, more fossil records are necessary to resolve the phytogeographic question of *Choerospondias*.

### 4.2. Paleoclimatic Implications of the Current Choerospondias Fossils

Extant *Choerospondias axillaris* is a deciduous tree that mainly grows in subtropical and tropical mountain forests with an elevation of 300–2000m, constituting minor components of subtropical to tropical evergreen forests in China [13,81,82,83]. Ye et al. [84] suggested that low temperature was a primary environmental factor influencing the distribution of the *Choerospondias* plant, followed by frost. Warm and humid climates are more favorable for the growth of *Choerospondias* [85]. Based on the living environments of extant *Choerospondias*, semiquantitative climatic parameters in the late Miocene eastern Zhejiang were obtained using the method of climate analysis of endemic species (CAES) [86,87], including the mean annual temperature (MAT) 5.7–24.7 °C, mean annual precipitation (MAP) 669–2435 mm, mean temperature of the warmest month (MTWM) 14.2–29.9 °C, and mean temperature of the coldest month (MTCM) −4.1–19.8 °C. To obtain more accurate MAT and MAP values, Ye et al. [84] used the MaxEnt model to reconstruct the two climatic values based on the distribution area of living *Choerospondias* plants, the MAT of 12.3 to 25.5 °C and the MAP of 950 to 2700 mm, respectively. The paleoclimatic parameters were further compared with the data reconstructed in previous studies. 

Many plant fossils from the Shengxian Formation in eastern Zhejiang have been identified, and their modern equivalents are mostly distributed in tropical or subtropical humid zones [24,25,88]. The MAT and MAP values in late Miocene eastern Zhejiang have been quantitatively reconstructed based on these plant fossils in previous studies (Table 3). Li [89] used the coexistence approach to reconstruct MAT as 16.3–20 °C and MAP as 1160.9–1653.5 mm based on plant macrofossils. Ding [90] used the Climate–Leaf Multivariate Analysis Program (CLAMP-ASIA1) to reconstruct MAT as 15.89 °C based on plant macrofossils. In this article, MAT and MAP were also reconstructed as 14.1–18.5 °C and 825.9–1470.2 mm, respectively, by overlapping distribution analysis. Yang [88] also reconstructed the MAT of 17.0–18.5 °C and the MAP of 979–1722 mm using the coexistence approach based on the Sporopollen fossils. Hua [91] reconstructed MAT as 23 °C based on the cell aspect ratio method for Chinese fir fossils, which was higher than that of other botanical methods. Recently, a geochemical method was established by Herbert et al. [92], who used the alkenone unsaturation method to reconstruct MAT as 21–28 °C based on marine fossil algae. This value is conspicuously higher than the MAT values reconstructed from other methods (Table 3). In the above paleoclimatic data, MAT is a common value. In addition, temperature is the primary factor for controlling the distribution of *Choerospondias* [85]. Thus, we only compared the MAT values reconstructed by different methods. Due to the lack of a statistical framework for the coexistence approach, this method is highly vulnerable to the vagaries of statistical outliers and exotic elements [93,94,95]. However, in this study, we only drew a comparison between different MAT values. It was found that the covered range of climatic parameters obtained herein was larger than that of previous paleoclimatic values based on botanical methods. This is possible because only one species of *Choerospondias* was used for the paleoclimatic reconstruction, resulting in lower accuracy. Overall, the current MAT values were similar to those obtained by other methods. However, the MAT reconstructed by the alkenone unsaturation method was relatively high. This may be due to the differences between land and marine plant materials. For the MAP, the values reconstructed by different methods were also generally analogical, although the current MAP values covered a larger range. Finally, the MAT and MAP in late Miocene eastern Zhejiang were also compared to current climatic data from the same region. It was concluded that the late Miocene climate in eastern Zhejiang was similar to or warmer and more humid than the modern climate, which is consistent with the views of previous studies [88,89,90].

## Figures and Tables

**Table 1:** Comparisons of Zhejiang *Choerospondias* endocarps with extant and other fossil species.

Species	Endocarp Shape	Length (mm)	Width (mm)	Outer Surface of the Endocarp	Number of Locules Per Fruit	Locality	Age	Source
*C. axillaris*	Obovoid	21–25	14–17	Longitudinal ridges and small pits	Predominantly 5, occasionally 3, 4, or 6	--	Modern	[34]
*C. sheppeyensis*	Obovoid	12–13	11–12.5	Longitudinal rows of pits	5	Sheppey, England	Early Eocene	[3,4,37]
*C. turovensis*	Elongate obovoid	9.5–12	5–9	Longitudinal rows of pits	5 or 6	Turow, Poland	Middle Miocene	[6]
*C. fujianensis*	Obovoid to ovoid	15.7–21.4	15.7–20.5	Longitudinal grooves and scattered pits	5 or 7	Fujian, China	Middle Miocene	[13]
*C. nanningensis*	Obovoid	15–21	13–17	Obscure ridges and few pits	Predominantly 5, occasionally 3, 4, or 6	Nanning, China	Late Oligocene	[12]
*C. axillaris* (Roxb.)	Obovoid	21–25	14–18	Pits arranged over the entire surface	5	Honshu, Japan; Kyushu, Japan	Late Miocene/Pleistocene	[8]
*C.* sp. cf. *C. axillaris*	Ovoid	45	23	no description	5	Honshu, Japan	Pliocene	[38]
*C. tiantaiensis* sp. nov.	Obovoid	16.4–20	9.8–11.7	Scattered, obvious pits; prominent bottom	5	Zhejiang, China	late Miocene	(This study)

**Table 2:** Comparison of leaf architecture and cuticular features of fossil and extant *Choerospondias*.

Type		Fossil *Choerospondias*	Extant *Choerospondias*
Leaf morphological characteristics	Leaf shape	Ovate to ovate-lanceolate, long acuminate apex, slightly convex base, broadly cuneate, asymmetrical at the basal insertion, petiole not preserved, leaf base angle about 100~119°	Ovate, ovate-lanceolate, long acuminate apex, broadly cuneate or subrounded base
	Leaf size	5–8.5 cm long, 2–3.6 cm wide	4–12 cm long, 2–4.5 cm wide
	Leaf edge	Untoothed margin	Untoothed margin or serrate in young plants
	Venation of leaves	Lateral veins arranged pinnately, 10 pairs, opposite or alternate; leaf apices at an angle of about 53°; simple brochidodromous, lateral veins at a more uniform angle with midrib, about 40–60°	Lateral veins pinnately arranged, Simple brochidodromous, 8~12 lateral veins on each side, lateral veins with midrib angle about 50–60°
Epidermal characteristics	Upper epidermis	Irregularly polygonal epidermal cells, varying from quadrilateral to hexagonal; shallowly undulated anticlinal wall	Polygonal epidermal cells; anticlinal walls slightly curved, with a few stomatal apparatuses
	Lower epidermis	Irregularly polygonal epidermal cells with shallowly undulated anticlinal walls, a trichome base, and multicellular roots	Polygonal epidermal cells with trichome bases, multicellular trichome roots, and radially arranged cells at the base of the trichome
	Stomatal apparatus	Cyclocytic stomatal apparatus, about 15–35 μm long and 10–30 μm wide; stomatal apparatus stellate distribution	The transition between anomocytic and cyclocytic, about 35–65 μm long and 24–50 μm wide

**Table 3:** Late Miocene climatic values reconstructed by different methods and nowadays values in Tiantai region, eastern Zhejiang.

Approach	MAT/°C	MAP/mm	Source
Coexistence approach	16.3–20	1160.9–1653.5	[89]
Palynoflora coexistence approach	17.0–18.5	979–1722	[88]
Overlapping distribution analysis	14.5–18.0	825.9–1470.2	[90]
CLAMP method	15.89	-	[90]
Cell aspect ratio method	23	-	[91]
Alkenone unsaturation method	21–28	-	[92]
Climatic factors for extant *Choerospondias* (CAES)	5.7–24.7 (12.3–25.5 *)	669–2435 (950–2700 *)	[87]
Nowaday climate of Tiantai	16.7	1391.5	[90]

* The values obtained by Ye et al. using the MaxEnt model [84].

## Data Availability

The data presented in this study are available on request from the corresponding author.
